# A personalized simulation framework for optimizing orthokeratology lens parameters based on 3D corneal modeling and biomechanical analysis

**DOI:** 10.3389/fbioe.2026.1869270

**Published:** 2026-07-24

**Authors:** Qi-ou Chen, Enxu Peng, Gaiping Zhao, Ping Ye, Zhaohua Chang

**Affiliations:** 1 School of Health Science and Engineering, University of Shanghai for Science and Technology, Shanghai, China; 2 College of Engineering, Zhejiang University, Hangzhou, Zhejiang, China

**Keywords:** biomechanics, finite element analysis, lens design, myopia control, orthokeratology, tear squeeze film

## Abstract

The aim of this study was to develop a patient-specific finite element model (FEM) to elucidate the biomechanical mechanisms governing orthokeratology (OK) lens design in real ocular conditions. Based on clinical corneal topography data, a patient-specific FEM was developed to incorporate asymmetric anatomical features. This model integrated an improved Iterative Closest Point (ICP) algorithm and a non-uniform squeeze-film pressure field. Simulation results were then compared with clinical measurements across multiple dimensions to evaluate predictive accuracy. The simulation framework demonstrated high predictive fidelity, The average Structure Similarity Index (SSIM) was 0.73, and the average Pearson correlation coefficient (PCC) reached 0.85. Treatment zone metrics: ( $TZ_{\mathrm{radius}}$ accuracy =93.7% , Mean Absolute Error, MAE=0.14 mm ); ( $TZ_{S}$ accuracy =88.8% , $MAE=1.73\;mm^{2}$ ). The maximum dioptric change ( $D_{\max}$ accuracy =79.0% , MAE=0.57D ) Statistical analysis was performed to evaluate the effects of two Back Optic Zone Diameters (BOZD) and two Targeted Dioptric Reductions (TDR) on biomechanical outcomes. A personalized FEM for the biomechanical analysis of OK was successfully developed, providing biomechanical theoretical support for the optimization of customized lens parameters.

## 1 Introduction

Global myopia in children and adolescents has risen from 24.32% in the 1990s to 35.81% in the early 2020s. It is projected to reach a prevalence of 39.80% by 2050, which is estimated to affect over 740 million young people worldwide (Liang et al., 2025). OK lenses are worn overnight to temporarily reshape the cornea. This provides clear daytime vision without aids and simultaneously manages myopia progression by creating a peripheral defocus (Wen et al., 2015; Tang et al., 2023; Fan et al., 2026). OK lenses have become the mainstay of clinical intervention for myopia control. Traditional OK lenses design does not accommodate individual corneal shape or blinking dynamics, leading to suboptimal outcomes like lens decentration and regression in about 30% of cases (Wang and Yang, 2019; Lin et al., 2024; Sun et al., 2022). Hence, developing a personalized biomechanical design theory for ortho-k is an urgent priority.

A key limitation in corneal biomechanical simulation is the oversimplified geometry used in most FEMs (Wu et al., 2021; Zhao et al., 2023; Zhang et al., 2025; Peng et al., 2025). These models typically rely on rotationally symmetric shapes (e.g., spheres, torics) to enforce regularity, which fails to capture the eye’s true asymmetric topology—such as temporal flattening and vertical curvature gradients—leading to distorted stress predictions.

This includes methods that reconstruct surfaces via low-order Zernike polynomial fitting (Wu et al., 2024) which smooth out high-order asymmetric features, resulting in an effectively rotationally symmetric corneal geometry. In such frameworks, any asymmetry in the simulation is introduced solely through externally applied conditions, not from the patient’s inherent anatomy. In Wu et al., 2024, lens decentration was measured clinically from topography first and then imposed into the FEA model as a known parameter. Additionally, By ignoring the 3D tear film gradient and its squeeze-film pressure, models that assume a uniform load create a biologically invalid simulation of the lens-cornea interface. The compromised accuracy of the registration algorithm prevents effective fusion of patient-specific modeling and clinical data. There is a lack of quantitative validation for the biomechanical effects of key parameters such as BOZD and TDR. This study establishes the first biomechanical simulation framework for personalized OK models, driven by *in vivo* human corneal topography. Key innovations include: 1) Accurate Modeling: Patient-specific 3D corneal geometries are reconstructed from clinical topography data, preserving key asymmetric features. 2)Enhanced Physical Mechanisms:This was achieved by incorporating an improved ICP algorithm (Besl and McKay, 1992; Pavlov et al., 2017; Zhang et al., 2020) for anatomically-precise lens-cornea registration, which addresses dynamic lens-wearing posture errors. Furthermore, a theoretical model of the non-uniform squeeze-film pressure field (Blech, 1983; Yin et al., 2006) was introduced to compute the blinking-induced pressure load based on the 3D interfacial gap distribution. 3) Multidimensional Validation and Mechanistic Analysis: The framework included morphological and optical metrics. The analysis also incorporated Cumulative Relative Corneal Refractive Power $\left(RCRP_{\mathrm{sum}}\right)$ in the Pupillary Area (Chen et al., 2025), a parameter directly predictive of OK treatment outcome (Wang et al., 2018; Lin et al., 2022; Li N. et al., 2025, Li et al., 2026). Subsequently, the resulting simulation data were used to elucidate the mechanistic influence of BOZD and TDR.

This study successfully developed a patient-specific FEM. The model demonstrated high predictive accuracy, achieving an SSIM>0.7 , a 93.7% accuracy in $TZ_{\mathrm{radius}}$ prediction, and an 88.8% accuracy in $TZ_{S}$ prediction. The model offers a methodological framework for conducting finite element analysis of the real cornea integrated with the tear film layer. It thereby offers a predictive tool to support clinical OK lens fitting and a methodological foundation for designing customized OK lenses.

## 2 Materials and methods

### 2.1 Clinical data and subject selection

Retrospective clinical data were retrieved from the fitting database of Shanghai MicroPort Vision Medical Technology Co., Ltd., with approval from the ethics committee of the Eye Hospital of Wenzhou Medical University (approval No. 2022.017) and in compliance with ethical principles (Gearing et al., 2006; Benchimol et al., 2015). To obtain a focused cohort, two strict inclusion criteria were applied: 1) a minimum lens wear duration of 1 month to ensure corneal shape stability, and 2) the exclusive use of standard spherical lens designs, thereby isolating the fundamental biomechanical effect by excluding toric lenses. Based on these criteria, a total of 35 cases of monocular corneal topography data were included as the foundation for personalized modeling. The baseline characteristics of the patients, along with the post-orthokeratology visual acuity and residual diopter measured after 1 month of lens wear, are summarized in Table 1.

**TABLE 1:** Basic Information Statistics of 35 eyes Included in This Study.

Statistical indicator	Mean ± sd	Range
Age (years)	25.14±9.99	10.00∼44.00
Spherical power(D)	−3.0±1.31	−4.00∼−1.00
Cylinder power(D)	−0.30±0.36	−1.00∼0.00
Corneal flat K value (D)	42.97±1.23	40.35∼45.08
Corneal ΔK (D)	0.65±0.38	0.10∼1.20
Post-ortho-K visual acuity	0.98±0.17	0.60∼1.20
Residual diopter(D)	0.16±0.39	−1.25∼1.00
Eye distribution	OD 19 eyes, OS 16 eyes	—

OD: right eye; OS: left eye; D: Diopter.

### 2.2 Construction of patient-specific 3D FEM

#### 2.2.1 Corneal geometric reconstruction

**FIGURE 1:**
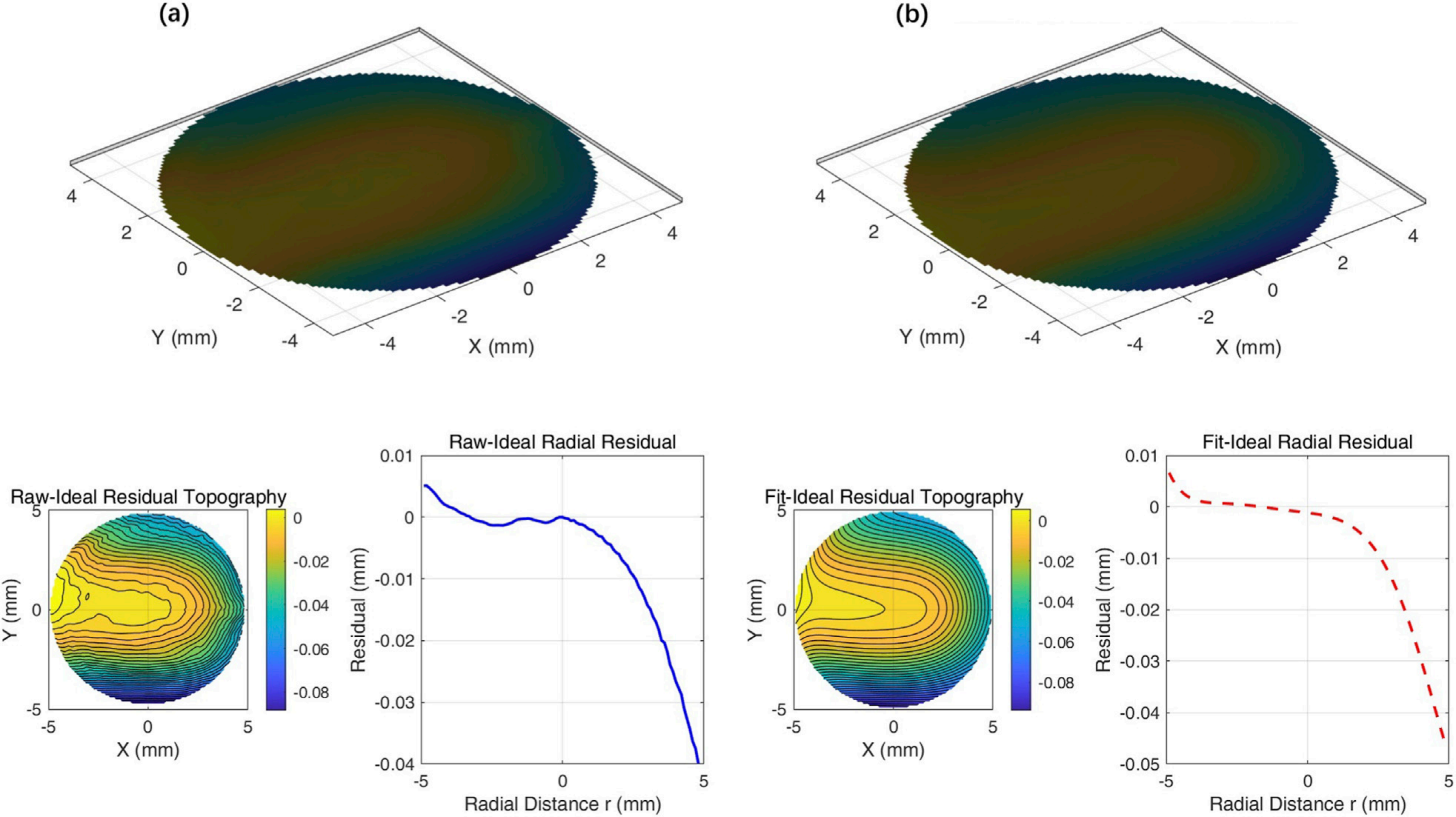
Preprocess raw data and Preserve key corneal information. (This process can capture the corneal anisotropic curvature, oblique astigmatism, and local asymmetry). **(a)** Residual: Raw Surface vs Ideal Surface. **(b)** Residual: 4th-Order Polynomial vs Ideal.

**FIGURE 2:**
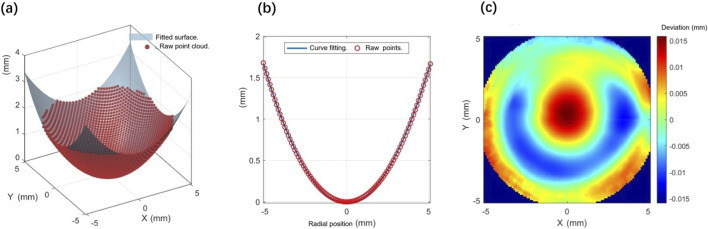
Data Preprocessing and Surface Fitting Validation. (The reconstruction process preserves key asymmetric features for personalized finite element modeling). **(a)** 3D reconstruction based on point clouds. **(b)** Central cross-sectional contour. **(c)** Fitted residual distribution plot.

A custom MATLAB program processed raw Medmont E300 corneal topographer data. Data preprocessing involved cleaning invalid points (NaN). Height and distance data were first gridded and reconstructed from scattered point clouds via cubic spline interpolation using the griddata function, and then converted to Cartesian coordinates (array size $101^{*}$ 101) (Subfig. 1(a)). Subsequently, a 4th-order polynomial surface is fitted in the Cartesian coordinate system. This polynomial incorporates quadratic terms $x^{2}$ , $y^{2}$ and the cross term xy with independent coefficients. Such terms enable flexible characterization of anisotropic curvature, oblique astigmatism and local asymmetric features, free from rotational symmetry and polar orthogonality constraints (Figure 1). The fitting strategy was chosen for its accuracy in representing core geometric features (e.g., astigmatism), optimal smoothness to avoid mesh distortion, and high fidelity (Root Mean Square Error, RMSE<0.001 mm ) (Figure 2). (Vahdati et al., 2016; Pang et al., 2024). The parameterized surface was imported into COMSOL Multiphysics (Version 6.0, COMSOL Inc., Sweden). A 3D solid corneal model was constructed by extruding the fitted anterior surface to form a spherical cap with a base radius of 5.1 mm. This diameter, approximating the average corneal diameter, was chosen to ensure the biomechanical effects of the OK lens were contained within the model domain without influence from distant boundary constraints. The cornea was modeled as a two-layer structure, comprising a 50 μ m epithelium and a 500 μ m stroma (Vahdati et al., 2016; Pang et al., 2024). This bilayer representation accounts for the significant stiffness difference between the layers, which is biomechanically critical for accurately simulating corneal reshaping.

#### 2.2.2 OK lens modeling

The lens geometry strictly followed the VST design principle (Li D. et al., 2025). It comprised four functional arcs from the center to the periphery: the Base Curve (BC), the Reverse Curve (RC), the Alignment Curve (AC)—which features an aspheric design distinct from segmented designs like AC1/AC2—and the Peripheral Curve (PC). For each patient, a parameterized 2D lens profile was constructed in COMSOL based on the actual fitting parameters (radius r , eccentricity e , and width w for each arc), ensuring smooth transitions between adjacent arcs. This composite curve was then revolved around the optical axis in a rotational sweep operation to generate the corresponding 3D solid lens model. A statistical summary of the lens fitting parameters is provided in Table 2. This study precisely registers the generated lens geometry onto the individual patient’s asymmetric 3D cornea using an improved ICP algorithm.

**TABLE 2:** OK lens Fitting Parameters Statistics (n = 35).

Statistical indicator	Mean ± sd	Range
Flat keratometry(D)	42.9857±1.21864	40.75∼45.25
Lens Diameter (mm)	10.463±0.2402	10.0∼10.8
TDR(D)	−3.3429±1.54359	−4.25∼−1.25
BOZD (mm)	5.954±0.3071	5.0∼6.6
$r_{BC}$	8.6716±0.44890	7.58∼9.57
$r_{RC}$	6.7570±0.18569	6.37∼7.04
$r_{AC}$	7.8576±0.22198	7.46∼8.28
$r_{PC}$	10.8281±0.48898	9.95∼11.58
$w_{BC}$	2.980±0.1530	2.5∼3.3
$w_{RC}$	0.6000±0.0000	0.60∼0.60
$w_{AC}$	1.1514±0.17677	0.90∼1.70
$w_{PC}$	0.5000±0.0000	0.50∼0.50
$e_{BC}$	1.1712±0.57470	0.00∼2.38
$e_{RC}$	0.00±0.000	0∼0
$e_{AC}$	0.500±0.0000	0.5∼0.5
$e_{PC}$	0.00±0.000	0∼0
Jessen factor	0.7500±0.00000	0.75∼0.75

Parameter definitions: $r_{BC}$ - $r_{PC}$ : BC - PC Radius; $w_{BC}$ - $w_{PC}$ : BC - PC Width; $e_{BC}$ - $e_{PC}$ : BC - PC Eccentricity.

#### 2.2.3 Lens-cornea registration via improved ICP algorithm

**FIGURE 3:**
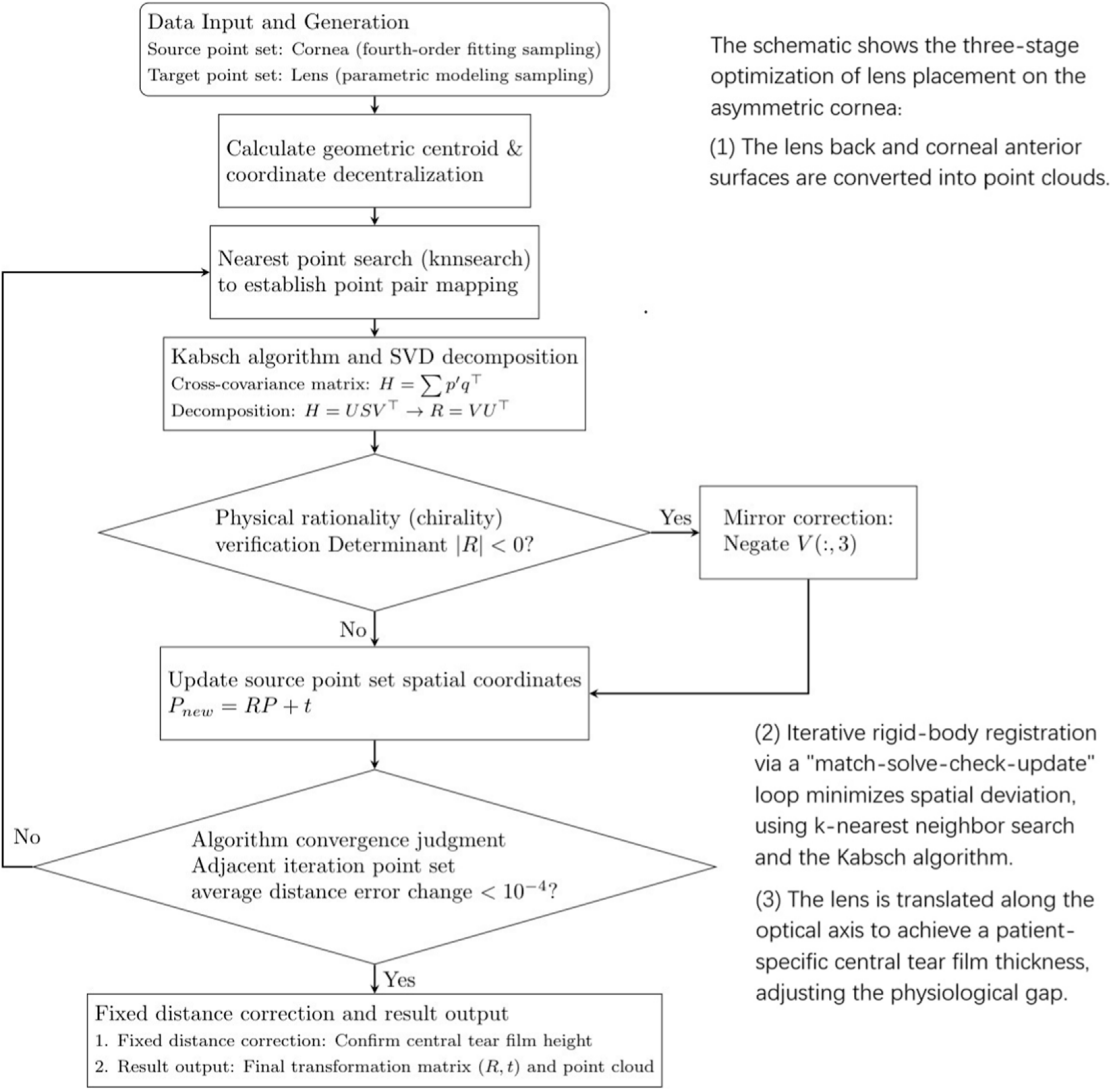
Flowchart of the Virtual Fitting Strategy Based on the Improved ICP Algorithm. (This workflow enables anatomically precise and stable lens-cornea alignment).

To determine the optimal position of the lens on the irregular corneal surface, an improved ICP algorithm was employed (Besl and McKay, 1992; Pavlov et al., 2017; Zhang et al., 2020). The key improvements over the standard ICP algorithm include: (1) the use of a k-nearest neighbors (knnsearch) algorithm for robust initial point correspondence matching, and (2) the implementation of a distance threshold to reject outlier point pairs during iteration, which enhances stability against surface noise and ensures biologically plausible alignment. This process consists of three main stages: point-cloud generation, iterative rigid-body registration, and physiological gap adjustment, as illustrated in Figure 3. The target point cloud was extracted from the rear surface of the lens model. The optimal lens position was then determined using an improved ICP algorithm that iteratively solves for the rigid-body transformation minimizing spatial deviation between the two point clouds via a “match-solve-check-update” loop

The computational procedure is described as follows: First, the k-nearest neighbors (knnsearch) algorithm (Halder et al., 2024) establishes point-to-point correspondence by matching each point on the back surface of the lens to its closest counterpart on the corneal surface based on Euclidean distance. Next, the Kabsch algorithm computes the optimal rigid transformation. The geometric centroids, $\bar{p}$ and $\bar{q}$ , are calculated for the lens point set $\left\{p_{1},p_{2},\ldots ,p_{N}\right\}$ and the matched corneal point set $\left\{q_{1},q_{2},\ldots ,q_{N}\right\}$ , respectively (Equation 1).

$$\bar{p}=\frac{1}{N}\overset{N}{\underset{i=1}{\sum }}p_{i},\;\bar{q}=\frac{1}{N}\overset{N}{\underset{i=1}{\sum }}q_{i}$$ (1)

Based on the computed centroids, both point sets are translated to a common origin. The decentralized vectors are defined as $p_{i}^{\prime }=p_{i}-\bar{p}$ and $q_{i}^{\prime }=q_{i}-\bar{q}$ . Subsequently, the cross-covariance matrix H is constructed by summing the outer products of all corresponding point pairs (Equation 2):

$$H=\overset{N}{\underset{i=1}{\sum }}p_{i}^{\prime }q_{i}^{\prime T}$$ (2)

The matrix is then subjected to singular value decomposition (SVD) (Equation 3).

$$H=U\Sigma V^{T}$$ (3)

The rotation matrix is then derived from the decomposition as $R=VU^{T}$ . To ensure physical plausibility, the transformation is validated. Since singular value decomposition can produce an improper rotation (reflection), the determinant |R| is checked. If |R|<0 , the third column of matrix V is negated to reconstruct a proper rotation matrix that satisfies the right-hand rule.

**FIGURE 4:**
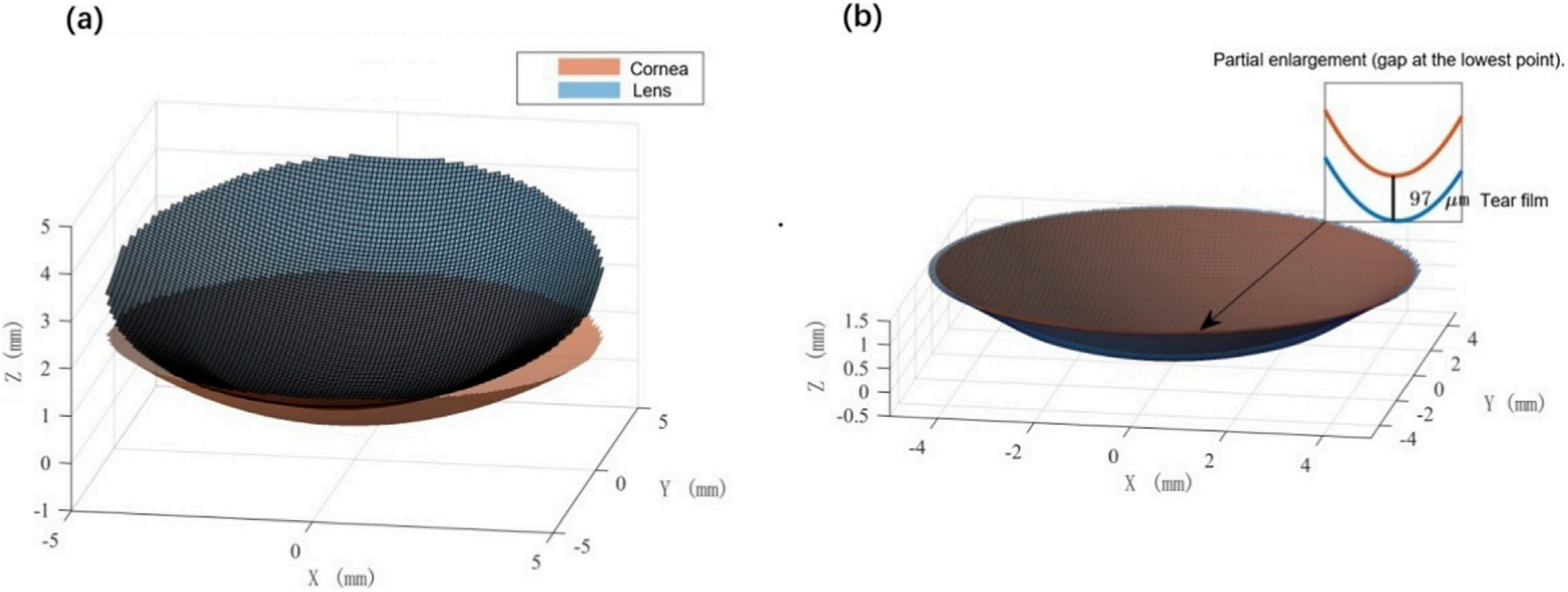
Illustration of the ICP Registration. (The registration achieves optimal alignment by minimizing the mean distance error between the lens back surface point cloud and the corneal surface point cloud). **(a)** Initial Position of the Cornea and Lens. **(b)** State of Optimal Alignment.

Finally, the source point cloud is updated using the corrected rotation matrix R and translation vector t . The process iterates until the mean distance error changes by less than $\left(1\times 10^{-4}\;mm\right)$ between iterations, indicating convergence. This achieves optimal lens-cornea alignment, as shown in Figure 4. Following ICP registration, the lens was translated along the optical axis (Z-axis) to ensure a physiological central tear film thickness. This thickness is defined as $h_{\mathrm{central}}=TDR+13\;\mu m$ , establishing a realistic tear layer in the model. Here, the 13 μm offset represents the nominal post-lens tear film thickness under a properly fitted lens, as supported by prior literature on tear film dynamics (King-Smith et al., 2000). This adjustment establishes a realistic baseline interfacial gap for the subsequent pressure calculation.

### 2.3 Mechanical loading and simulation configuration

#### 2.3.1 Non-uniform squeeze film pressure field

A patient-specific pressure field $F_{\mathrm{map}}\left(x,y\right)$ was constructed based on the 3D tear film distribution h(x,y) . First, the theoretical maximum squeeze film pressure $P_{\text{max}}$ was calculated using the classic fluid mechanics solution for parallel plate squeeze film (Blech, 1983): (Equation 4).

$$P_{\max}=\frac{3\mu VD^{2}}{8h_{0}^{3}}\cdot \frac{2-\alpha }{{\left(1-\alpha \right)}^{2}}$$ (4)

Here, $\alpha =\frac{h_{0}-h_{c}}{h_{0}}$ is a dimensionless parameter representing the relative change in tear film thickness. The following fluid and kinematic parameters were used: viscosity μ=0.0008 Pa·s (King-Smith et al., 2000) (for normal tear fluid); eyelid-closure speed V=0.01 m/s (representing the average blink-approach velocity) (Doane, 1980); and effective diameter D=10.6 mm , corresponding to the standard OK lens landing-zone diameter (Swarbrick et al., 1998).

A Sigmoid function served as the spatial modulation operator, defining a normalized scaling factor λ(x,y) that maps the actual tear-film height distribution h(x,y) to the interval [0,1] (Equation 5).

$$\lambda \left(x,y\right)=\frac{h\left(x,y\right)-h_{c}}{h_{0}-h_{c}}$$ (5)

Development of the Basic Sigmoid Modulation Function S(λ) (Equation 6):

$$S\left(\lambda \left(x,y\right)\right)=\frac{1}{1+e^{-k\left(\lambda \left(x,y\right)-x_{0}\right)}}$$ (6)

Here, k and $x_{0}$ are the key parameters controlling the pressure distribution shape. The steepness factor k was set to 5, ensuring a physically appropriate smoothness for the function. The center threshold $x_{0}$ was determined as 0.25 via numerical inversion, based on the finding that the effective pressure zone (normalized pressure >0.5 ) occupies about 36% of the radial width in the standard axisymmetric model.

Based on the calculated physical peak pressure and spatial weighting, data cleaning and spatial domain limitation were applied to synthesize a 3D non-uniform squeeze-film pressure field $F_{\mathrm{map}}\left(x,y\right)$ acting on the corneal surface (Equation 7).

$$F_{\mathrm{map}}\left(x,y\right)=P_{\max}\cdot S\left(\lambda \left(x,y\right)\right)$$ (7)

#### 2.3.2 Boundary conditions and material properties

Boundary conditions were set by modeling eyelid pressure as a uniform, downward vertical load on the anterior corneal surface. Its magnitude is dynamically adjusted and varies linearly with the targeted dioptric reduction (TDR) (Equation 8).

$$P_{\mathrm{lid}}=-TDR\times 25+125$$ (8)

This relationship was derived from preliminary data fitting in our research, aiming to establish a quantitative link between the clinical fitting parameter (TDR) and the mechanical load applied in the simulation, representing the aggregate effect of eyelid pressure during sleep. At the cornea-sclera junction, all translational degrees of freedom were constrained by setting the displacements to zero. The OK lens, fabricated from Boston XO2 material, was defined as a linear elastic material with a Young’s modulus of 1160 MPa , while the corneal tissue was represented by a Neo-Hookean hyperelastic model (Seven et al., 2020), with the epithelium and stroma assigned shear moduli of 0.085 MPa and 0.85 MPa , and bulk moduli of 85 MPa and 850 MPa , respectively.

#### 2.3.3 Meshing

**FIGURE 5:**
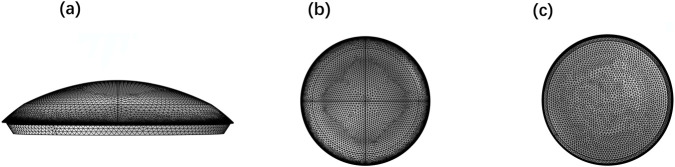
FEM Mesh Schematic. (A targeted meshing strategy was employed, with refined element density in the critical lens-cornea contact interface and treatment zone region to ensure computational accuracy for stress and deformation analysis). **(a)** Front View. **(b)** Top View. **(c)** Bottom View.

A targeted meshing strategy was employed, with the final mesh quality for the global model and key areas demonstrated in Figure 5. The geometry was discretized using an unstructured tetrahedral mesh.

#### 2.3.4 Mesh independence verification

A mesh sensitivity analysis was conducted to ensure that the simulation results were independent of the discretization density. For three representative patient models, a convergence study was performed by progressively refining the global and interfacial mesh, generating four mesh configurations from “coarse” to “extremely fine.” The key output variables for assessing convergence were the central corneal stress $\left(S_{\mathrm{center}}\right)$ , the maximum dioptric change $\left(D_{\max}\right)$ , and the central corneal displacement $\left(U_{\mathrm{center}}\right)$ . Convergence was assumed when the relative change in these variables between two successive mesh refinements was less than 2%. The analysis confirmed that the “fine” mesh configuration (as visualized in Figure 5) met this criterion, with changes in all key variables below 1.5% upon further refinement. Therefore, this mesh configuration was adopted for all subsequent simulations, ensuring an optimal balance between computational accuracy and efficiency.

### 2.4 Evaluation metrics and statistical analysis

The simulated corneal power change map was compared with clinically measured post-operative topography. Full-field metrics included: SSIM , PCC and RMSE . Key morphological parameters included: $TZ_{\mathrm{radius}}$ , $TZ_{S}$ , $D_{\max}$ and the value and position of the maximum dioptric change $\left(D_{\mathrm{maxPos}}\right)$ . Subjects were grouped by TDR (High: ≤−3.00D /Low: >−3.00D ) and BOZD (Large: ≥6.0 mm /Small: <6.0 mm ). Normality and homoscedasticity assumptions for all response variables (e.g., strain, stress, refractive changes, $RCRP_{\mathrm{sum}}$ ) were formally assessed using the Shapiro-Wilk tests, respectively. As these parametric assumptions were violated, the non-parametric Mann-Whitney U test was employed for all inter-group comparisons (Mann and Whitney, 1947). The effect size for each comparison was quantified using the non-parametric $\eta^{2}$ , calculated from the test’s Z-statistic $\left(\eta^{2}=Z^{2}/\left(N-1\right)\right)$ . This measure is robust to non-normal distributions and sample size imbalance.

## 3 Results

### 3.1 Validation of the patient-specific simulation model

**FIGURE 6:**
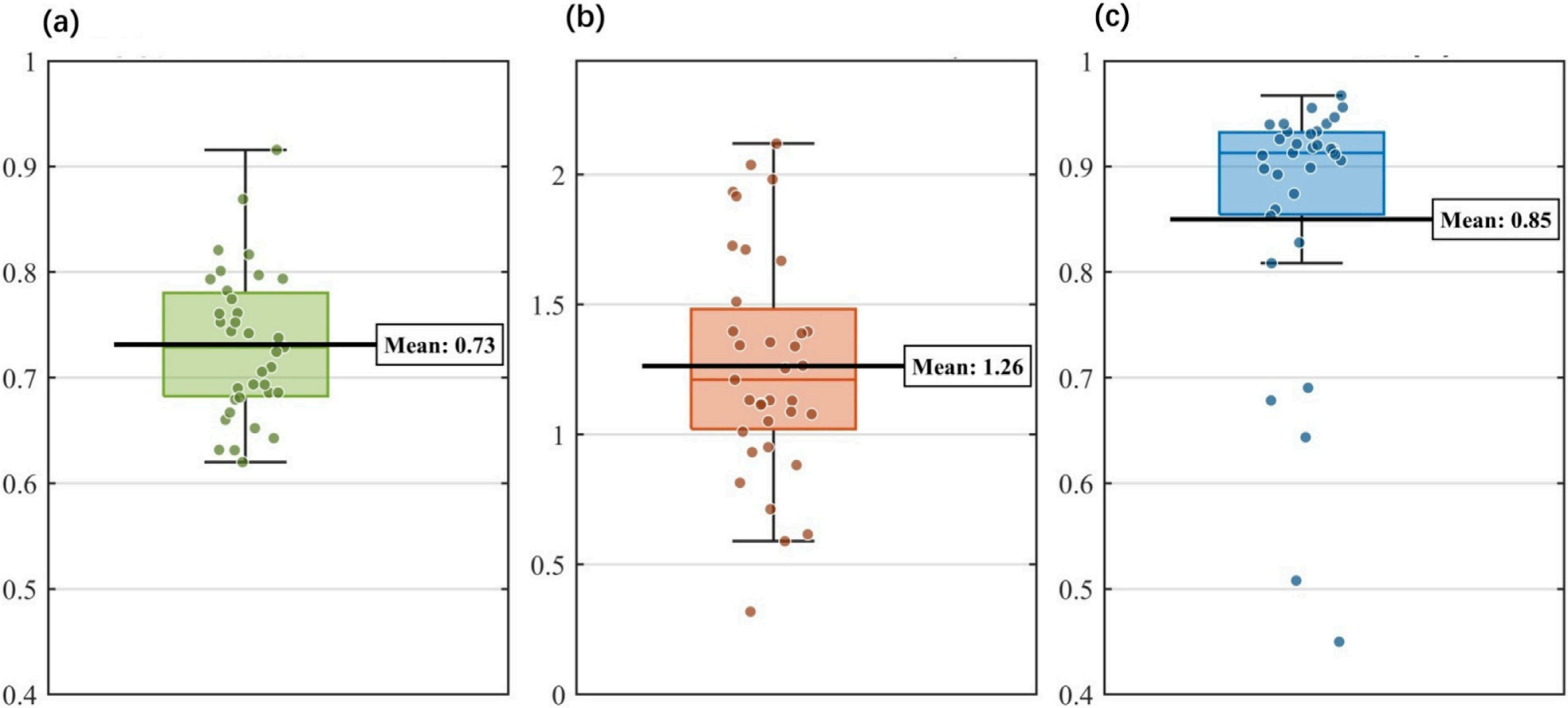
Statistical Distribution of Simulation Accuracy (Comparison between simulated and clinically measured post-orthokeratology corneal dioptric change maps). **(a)** SSIM. **(b)** RMSE, μm. **(c)** PCC.

The simulation system demonstrated high fidelity in predicting post-Ortho-K corneal reshaping. The average SSIM between simulated and clinical dioptric change maps was 0.73, indicating high morphological reproducibility. The average PCC was 0.85, demonstrating excellent agreement in spatial distribution trends (Figure 6). The average RMSE was 1.26D , with the deviation attributed primarily to the use of uniform material properties. The model showed high accuracy in predicting key morphological parameters: the prediction accuracy for $TZ_{\mathrm{radius}}$ was 93.7% (MAE=0.14 mm) , and for $TZ_{S}$ was 88.8% $\left(MAE=1.73\;mm^{2}\right)$ (Table 3).

**TABLE 3:** Statistical results of prediction errors for key clinical morphological parameters.

Result indicator	MAE	Accuracy (%)	Mean deviation
$TZ_{\mathrm{radius}}\;\left(mm\right)$	0.14	93.7	−0.05
$TZ_{S}\;\left(mm^{2}\right)$	1.73	88.8	−0.50
$D_{\max}\;\left(D\right)$	0.57	79.0	0.54

**FIGURE 7:**
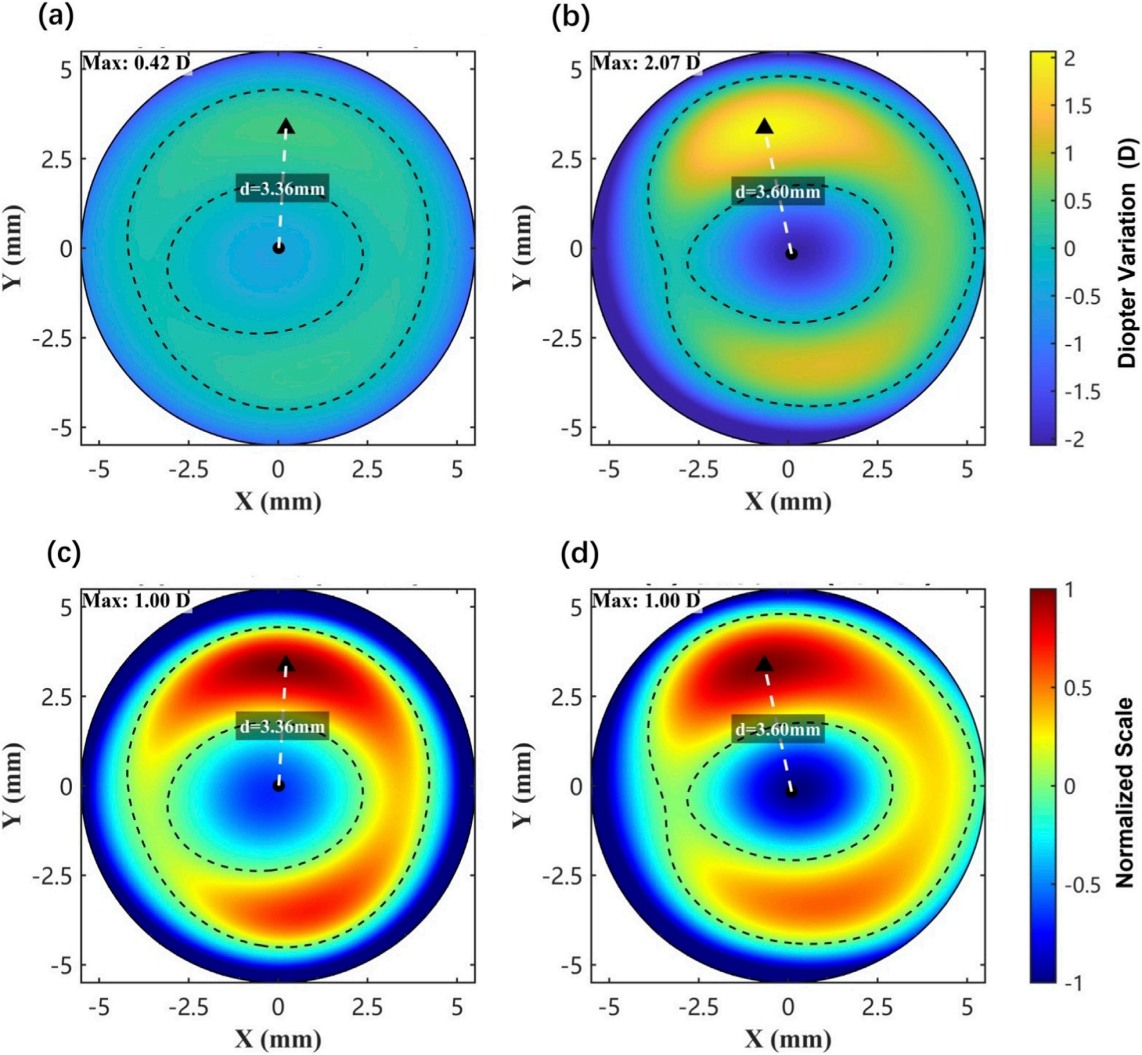
Simulated vs. Clinical Dioptric Difference Distributions (The high SSIM (0.73) and PCC (0.85) indicate the model’s accuracy in predicting both the magnitude and pattern of corneal reshaping). **(a)** Simulated dioptric change map. **(b)** Clinically measured dioptric change map. **(c)** Simulation (normalized) map. **(d)** Clinical measurement (normalized) map.

As shown in Figure 7, subfigures (a) and (b) present the raw dioptric change distributions from simulations and clinical measurements, respectively, to evaluate numerical accuracy. In contrast, subfigures (c) and (d) display the same distributions after sign-separated normalization (where positive and negative values are divided by their respective absolute maxima), in order to focus on morphological comparison by removing the influence of absolute magnitude differences.

### 3.2 Personalized 3D tear distribution construction results

Through the workflow in Figure 3, this study successfully obtained a personalized 3D tear film distribution image for each eye. Key statistical metrics are summarized in Table 4, quantifying the fluid gap heterogeneity induced by irregular corneal surfaces. This provides the essential data foundation for the subsequent calculation of a non-uniform squeeze-film pressure field (Equation 7).

**TABLE 4:** Summary of motion and tear film thickness statistics for all cases.

Statistical metric	Mean ± sd	Max	Min
Total rotation angle (°)	0.22 ± 0.083	0.44	0.036
Total translation displacement (mm)	0.079 ± 0.016	0.12	0.042
Central corneal tear thickness (mm)	0.0098 ± 0.0018	0.015	0.0063
Maximum corneal tear thickness (mm)	0.10 ± 0.016	0.14	0.07

**FIGURE 8:**
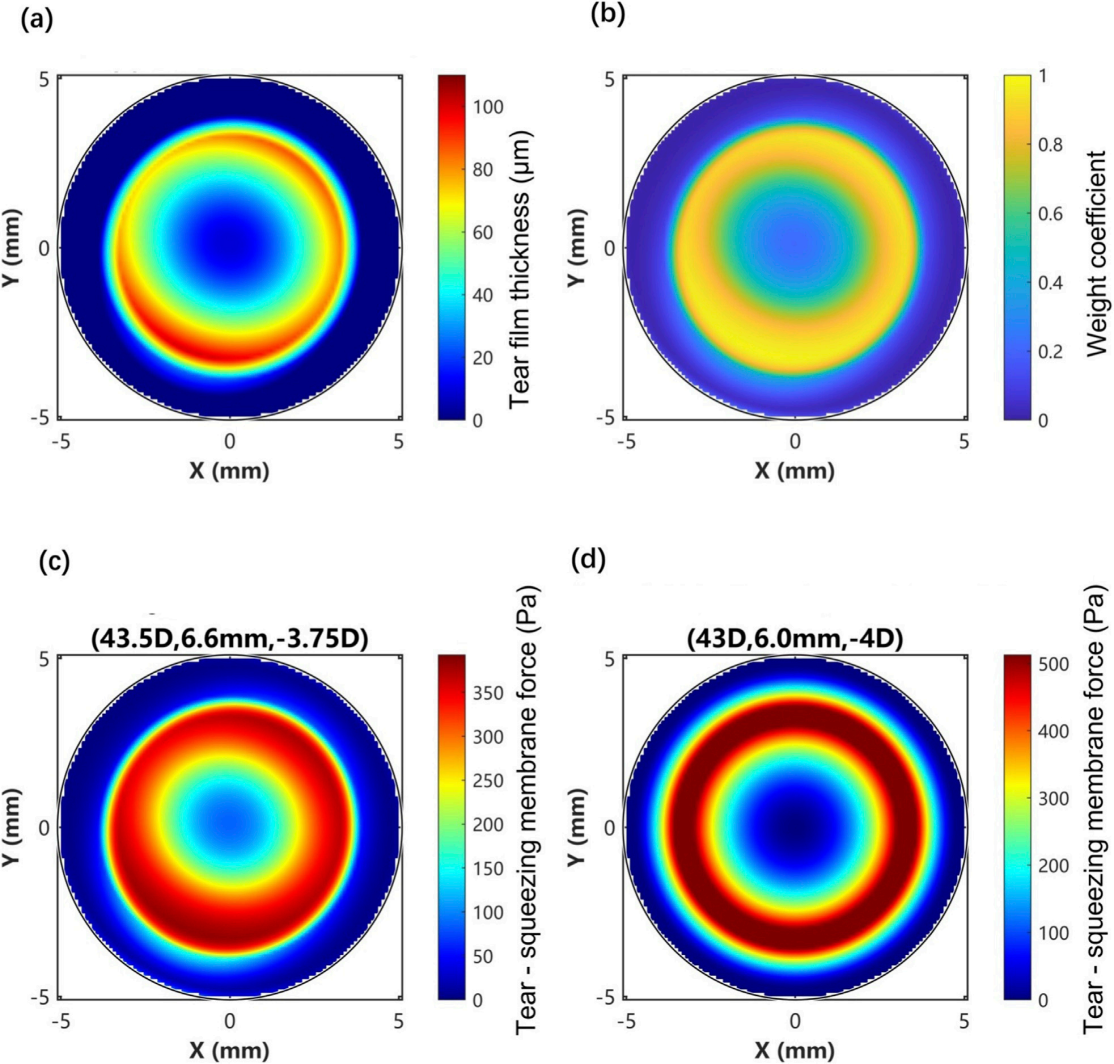
Methodology and a Comparative Study of the Non-Uniform Pressure Field (**(a)** The 3D tear film thickness distribution h(x,y) extracted from the lens-cornea interface after ICP registration. **(b)** The spatial weighting field λ(x,y) generated using a Sigmoid modulation function based on h(x,y) . **(c)** The synthesized personalized pressure field $F_{\mathrm{map}}\left(x,y\right)$ , obtained by multiplying the theoretical maximum pressure $P_{\max}$ with the weighting field. **(d)** For comparison, a traditional rotationally symmetric pressure model yields an idealized annular profile (Peng et al., 2025)). **(a)** Spatial distribution of tear height. **(b)** Distribution of the weight - function of tear - squeezing membrane force. **(c)** Distribution of the tear-squeezing membrane force field. **(d)** Distribution of the squeezing - membrane force field for the axisymmetric model Weight coefficient.

Figures 8a–c illustrate the algorithm’s process: extracting tear film thickness (a), generating a spatial weighting field (b), and synthesizing a personalized, non-uniform pressure field by multiplying with $P_{\max}$ (c). Comparison with the traditional rotationally symmetric model (d) (Peng et al., 2025) reveals a key difference: the conventional model yields an idealized annular pressure, masking corneal asymmetry. In contrast, our model accurately preserves asymmetric features, clearly revealing localized stress concentrations induced by surface irregularities. This significant stress heterogeneity underscores the necessity of moving beyond idealized assumptions for accurate mechanical prediction.

**FIGURE 9:**
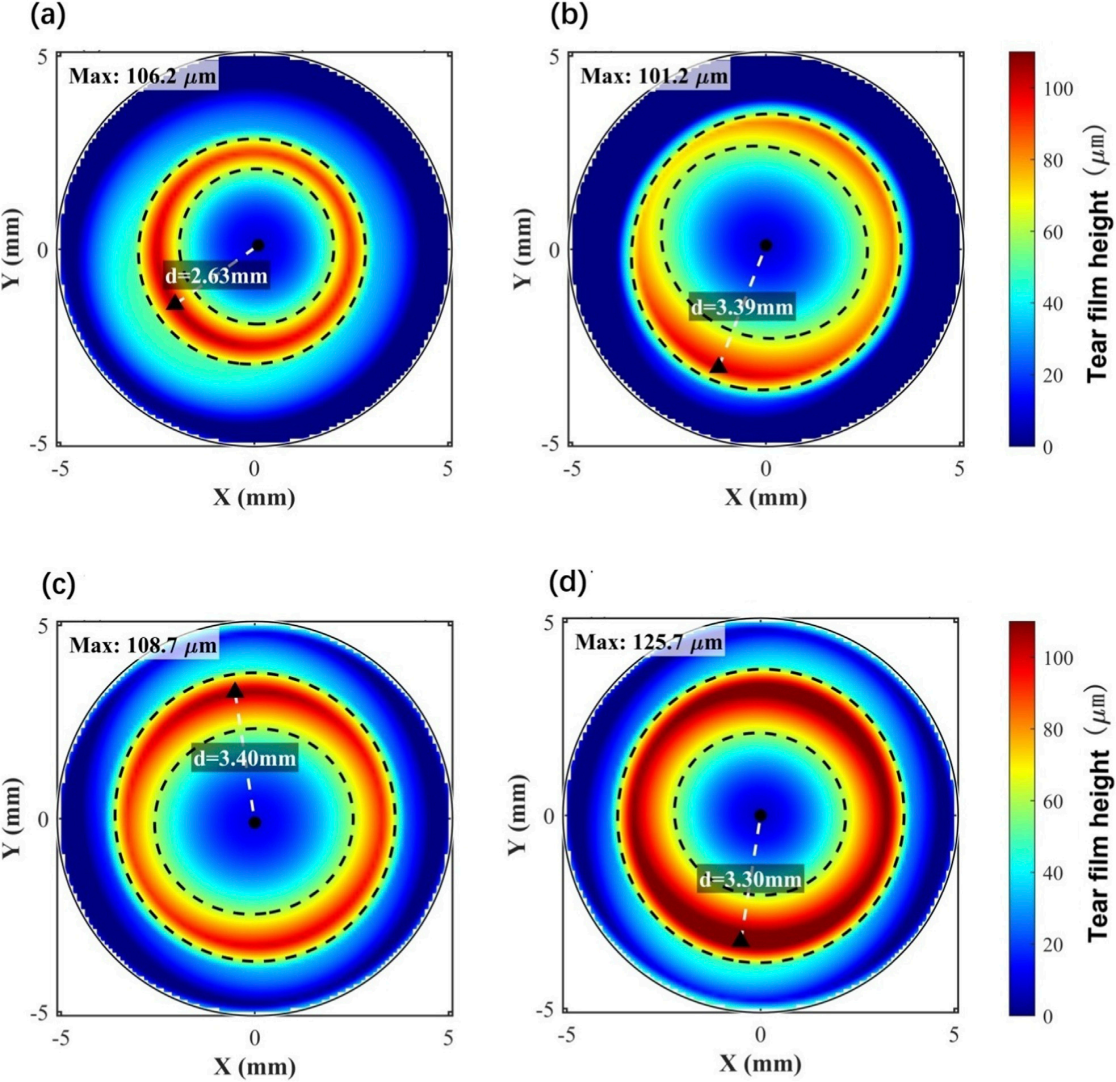
Impact of Key Design Parameters on Personalized Tear Distribution Morphology (**(a,b)** With a fixed TDR (−3.8D) , increasing BOZD from 5.0 mm to 6.6 mm causes a significant outward shift in the peak fluid reservoir location d , while its $h_{\max}$ remains stable **(c,d)** With a fixed BOZD (6.6 mm) , increasing the TDR (from −2.0D to −4.0D ) significantly increases the $h_{\max}$ , with minimal change in its position d . This confirms the fundamental design principles: BOZD primarily controls the position of the fluid reservoir, while TDR controls its depth). **(a)** BOZD = 5.0 mm (TDR = -3.8). **(b)** BOZD = 6.6 mm (TDR = -3.8). **(c)** TDR = −2.0*D* (BOZD = 6.6 mm). **(d)** TDR = −4.0*D* (BOZD = 6.6 mm).

A detailed comparison reveals distinct control effects of the two key design parameters. In Figures 9a,b, with a fixed TDR (−3.8D) , increasing the BOZD from 5.0 mm to 6.6 mm causes a significant outward shift in the peak location d (from 2.63 mm to 3.39 mm ), while the maximum thickness $h_{\max}$ remains stable. Conversely, in subfigures (c) and (d), with a fixed BOZD (6.6 mm) , increasing the TDR (from −2.0D to −4.0D ) significantly increases $h_{\max}$ ( 108.7 μm to 125.7 μm ), with minimal change in d . These results confirm that even for corneas with complex astigmatism, the fundamental physical rules hold: BOZD primarily controls the position of the fluid reservoir, while TDR controls its depth. This consistency not only validates the VST design theory but also demonstrates the reliability of our 3D model in characterizing lens-cornea interaction.

### 3.3 Biomechanical effects of design parameters

#### 3.3.1 Effect of TDR

The High-TDR group showed significantly higher central stress $\left(S_{\mathrm{center}}\right)$ , maximum stress $\left(S_{\max}\right)$ , central dioptric change $\left(D_{\mathrm{center}}\right)$ , and $D_{\max}$ compared to the Low-TDR group (all p<0.001 , Table 5). However, no significant difference was found in central or maximum axial displacement ( $U_{\mathrm{center}}$ , $U_{\max}$ ) between groups (p<0.05) .

**TABLE 5:** Comparison of simulation results under different TDR groups.

Evaluation index	Low-TDR group (n=17)	High-TDR group (n=18)	p -value
$U_{\mathrm{center}}\;\left(\mu m\right)$	6.224±1.065	6.374±1.248	0.705
$U_{\max}\;\left(\mu m\right)$	6.282±1.040	6.506±0.957	0.513
$U_{\mathrm{maxPos}}\;\left(mm\right)$	0.174±0.187	0.177±0.249	0.965
$S_{\mathrm{center}}\;\left(Pa\right)$	1831.743±204.048	2838.769±719.808	<**0.001**
$S_{\max}\;\left(Pa\right)$	1528.071±245.120	2217.891±451.784	<**0.001**
$S_{\mathrm{maxPos}}\;\left(mm\right)$	2.875±0.458	2.795±0.218	0.513
$D_{\mathrm{center}}\;\left(D\right)$	−0.736±0.194	−1.374±0.348	<**0.001**
$D_{\max}\;\left(D\right)$	6.441±0.943	8.124±1.531	<**0.001**
$D_{\mathrm{maxPos}}\;\left(mm\right)$	4.806±0.248	4.827±0.234	0.792
$TZ_{\mathrm{radius}}\;\left(mm\right)$	2.168±0.238	2.049±0.170	0.098
$RCRP_{\mathrm{sum}}\;\left(D\cdot mm^{2}\right)$	−4.909±1.614	−6.325±2.115	<**0.05**

#### 3.3.2 Effect of BOZD

The Large-BOZD group had a significantly larger $TZ_{\mathrm{radius}}$ ( 2.158±0.179 mm vs. 1.799±0.108 mm , p<0.05 ) and unexpectedly higher Scenter and Dcenter ( p<0.05 , Table 6) compared to the Small-BOZD group. However, it is important to note that the statistical comparisons involving the Small-BOZD group are based on a limited sample size. In contrast to findings from axisymmetric models, the $S_{\mathrm{maxPos}}$ and $D_{\mathrm{maxPos}}$ showed no significant difference between BOZD groups (p>0.05) .

**TABLE 6:** Comparison of simulation results under different BOZD groups.

Evaluation index	Large-BOZD (n=30)	Small-BOZD (n=5)	p -value
$U_{\mathrm{center}}\;\left(\mu m\right)$	6.517±0.692	5.006±2.299	0.132
$U_{\max}\;\left(\mu m\right)$	6.543±0.678	5.520±1.968	0.245
$U_{\mathrm{maxPos}}\;\left(mm\right)$	0.105±0.051	0.597±0.355	<**0.001**
$S_{\mathrm{center}}\;\left(Pa\right)$	2473.014±720.983	1609.407±131.693	<**0.05**
$S_{\max}\;\left(Pa\right)$	1965.054±494.338	1389.522±145.919	<**0.05**
$S_{\mathrm{maxPos}}\;\left(mm\right)$	2.878±0.259	2.569±0.684	0.163
$D_{\mathrm{center}}\;\left(D\right)$	−1.140±0.413	−0.608±0.128	<**0.05**
$D_{\max}\;\left(D\right)$	7.395±1.602	6.775±0.841	0.408
$D_{\mathrm{maxPos}}\;\left(mm\right)$	4.840±0.203	4.678±0.391	0.345
$TZ_{\mathrm{radius}}\;\left(mm\right)$	2.158±0.179	1.799±0.108	<**0.05**
$RCRP_{\mathrm{sum}}\;\left(D\cdot mm^{2}\right)$	−6.225±1.100	−2.109±2.622	<**0.05**

Caution is advised when interpreting p-values and effect sizes for the Small-BOZD group comparisons, as the limited sample size may reduce statistical power and increase result variability.

#### 3.3.3 Parameter control weights in the personalized context

Effect size $\left(\eta^{2}\right)$ analysis revealed a substantial re-weighting of parameter influence in real ocular surface environments (Table 7). TDR remained the dominant factor for intensity metrics ( $\eta^{2}=0.550$ for $S_{\mathrm{center}}$ , 0.567 for $D_{\mathrm{center}}$ ). BOZD effectively controlled $TZ_{\mathrm{radius}}$ ( $\eta^{2}=0.368$ ),but showed weak control over $S_{\mathrm{maxPos}}$ $\left(\eta^{2}=0.132\right)$ and $D_{\mathrm{maxPos}}$ ( $\eta^{2}=0.026$ ). Crucially, the $RCRP_{\mathrm{sum}}$ , the dominant factor shifted from TDR in axisymmetric models to BOZD in personalized models $\left(\eta^{2}=0.368\right)$ ,Compared with previous literature (Peng et al., 2025). A global attenuation of $\eta^{2}$ values for all metrics was observed, indicating the significant interfering effect of individual anatomical variation.

**TABLE 7:** Nonparametric tests and effect size results of core evaluation indicators.

Evaluation indicator	Dominant factor	$\eta^{2}$	Mean difference comparison
$U_{\mathrm{center}}\;\left(\mu m\right)$	BOZD	**0.032**	Large-BOZD vs. Small-BOZD: Δ=0.001511
$U_{\max}\;\left(\mu m\right)$	BOZD	**0.026**	Large-BOZD vs. Small-BOZD: Δ=0.001023
$U_{\mathrm{maxPos}}\;\left(mm\right)$	BOZD	**0.368**	Large-BOZD vs. Small-BOZD: Δ=−0.4921
$S_{\mathrm{center}}\;\left(Pa\right)$	TDR	**0.550**	Low-TDR vs. High-TDR: Δ=−1007
$S_{\mathrm{center}}\;\left(Pa\right)$	TDR	**0.542**	Low-TDR vs. High-TDR: Δ=−689.8
$S_{\mathrm{maxPos}}\;\left(mm\right)$	BOZD	**0.132**	Large-BOZD vs. Small-BOZD: Δ=0.3093
$D_{\mathrm{center}}\;\left(D\right)$	TDR	**0.567**	Low-TDR vs. High-TDR: Δ=0.6387
$D_{\max}\;\left(D\right)$	TDR	**0.388**	Low-TDR vs. High-TDR: Δ=−1.683
$D_{\mathrm{maxPos}}\;\left(mm\right)$	BOZD	**0.026**	Large-BOZD vs. Small-BOZD: Δ=0.1621
$TZ_{\mathrm{radius}}\;\left(mm\right)$	BOZD	**0.368**	Large-BOZD vs. Small-BOZD: Δ=−4.116
$RCRP_{\mathrm{sum}}\;\left(D\cdot mm^{2}\right)$	BOZD	**0.368**	Large-BOZD vs. Small-BOZD: Δ=0.3592

## 4 Discussion

This study successfully established a clinical topography-driven, patient-specific biomechanical simulation framework for orthokeratology. This study’s model can automatically achieve accurate lens registration and predict decentration via the improved ICP algorithm and non-uniform pressure field without prior clinical measurement. The model demonstrated high predictive accuracy ( SSIM=0.73 , PCC=0.85 , $TZ_{\mathrm{radius}}$ accuracy =93.7% ) (Table 3) (Figure 6). This marks the first time that the biomechanical effects of BOZD and TDR have been quantitatively analyzed in an asymmetric, real corneal geometry. The results reveal that individualized anatomical structures significantly alter the weighting of parameter influences. This work thereby bridges the critical gap between clinical data, precise biomechanical simulation, and personalized lens design.

The high model accuracy and clinical consistency, evidenced by SSIM , PCC , and MAE metrics, demonstrate the efficacy of the enhanced ICP registration and non-uniform pressure model in improving simulation realism. Unlike traditional symmetric FEMs, which fail to replicate clinical topography, our model preserved key asymmetric features (e.g., temporal flattening, vertical curvature gradients), achieving closer alignment with real ocular geometry. However, error sources remain, including the assumptions of material homogeneity, the neglect of corneal viscoelasticity, and diurnal dynamic variations. Addressing these limitations will be the focus of future work. This is consistent with recent research findings (Wu et al., 2021; Zhang et al., 2025; Peng et al., 2025; Lin et al., 2022; Li et al., 2023), TDR acts as the primary regulator of reshaping intensity: a higher value reduces the central tear film gap, increasing squeeze-film pressure to drive a stronger effect. Consequently, TDR primarily regulates central and maximum stress values, while concomitantly achieving significant $D_{\max}$ (Swarbrick et al., 1998; Cho and Cheung, 2012; Santodomingo-Rubido et al., 2012; Nti and Berntsen, 2020), and $RCRP_{\mathrm{sum}}$ . A smaller BOZD yields a smaller TZ, as consistently demonstrated in clinical and modeling studies (Guo et al., 2023; Li H. et al., 2025). A larger BOZD spreads this force, creating a wider but shallower reshaping zone (Zhang et al., 2025). While conventional symmetric models consider BOZD primarily for lens centration, our patient-specific asymmetric model reveals that BOZD may also be a dominant regulator of $TZ_{\mathrm{radius}}$ and $RCRP_{\mathrm{sum}}$ (Table 6).

In this study’s model,OK lens decentration arises naturally from the interaction between the asymmetric corneal geometry (e.g., temporal flattening, corneal astigmatism) and the improved ICP registration algorithm, which finds the optimal “fit” position rather than forcing central alignment. This mimics the clinical scenario where the OK lens settles into a position of minimal interfacial pressure imbalance. Our framework provides a tool to predict the amount and direction of decentration based on preoperative topography. Whether it can be stably controlledis a complex clinical question. The simulation suggests that by adjusting lens parameters (e.g., BOZD, alignment curve design) within the model, one could potentially influence the final lens position and the resulting defocus ring profile.

The innovations of this study lie in four aspects: 1) The clinical topography driven asymmetric 3D corneal modeling breaks through the conventional rotationally symmetric assumption. 2) The improved ICP algorithm enables precise lens-cornea registration and resolves the issue of non-centered lens positioning. 3) The non-uniform squeeze-film pressure field realistically reproduces the mechanical loading induced by blinking. 4)For clinical value, the proposed framework can serve as a preoperative prediction tool for clinicians, support the personalized optimization of OK lens parameters, and has the potential to improve fitting success rates, reduce lens decentration, and enhance myopia control efficacy.

This study has several limitations. 1) The overall sample size was limited to 35 cases, and corneas with high astigmatism were excluded, as only rotationally symmetric lens designs were modeled. 2) Notably, the sample size within the Small-BOZD subgroup was particularly small. This constraint directly impacts the statistical power and reliability of the comparative analyses involving BOZD. 3) the model uses a quasi-static hyperelastic formulation for the cornea and treats the Ortho-K lens as linear elastic. It ignores time-dependent viscoelastic behaviors of both corneal tissue (e.g., creep and stress relaxation) and lens materials. Since these characteristics are closely related to lens wearing duration and long-term corneal remodeling, the current model only describes the initial stress distribution induced by mechanical loading. Future models incorporating viscoelastic constitutive relations and dynamic wear cycles are needed to fully capture the time-varying treatment effects. 4) The OK lens was modeled as a linear elastic material. While this is a valid simplification for the primary biomechanical analysis presented, future models could incorporate hyperelastic or viscoelastic material laws for the lens to investigate more complex dynamic interactions. 5) The analysis was restricted to static mechanics, not simulating the full dynamic process of overnight wear. 6) Individual variations in eyelid pressure and tear film rheology were not considered. Future research will focus on the following steps: to expand the cohort into a multicenter model, to implement 4D dynamic spatio-temporal simulations, to integrate AI for automated parameter optimization, and to initiate prospective clinical trials for validating the simulation-guided fitting protocol.

This personalized biomechanical simulation framework provides a reliable and quantitative tool for understanding OK mechanisms and optimizing lens parameters toward truly customized myopia control.

## Data Availability

The clinical dataset supporting this study is not publicly available due to patient privacy and ethical restrictions. Data access may be granted to qualified researchers upon reasonable request to the corresponding author and with approval from the Institutional Review Board of School of Health Science and Engineering. Requests should include a detailed research proposal and data protection plan.
